# miR-135a-5p overexpression in peripheral blood-derived exosomes mediates vascular injury in type 2 diabetes patients

**DOI:** 10.3389/fendo.2023.1035029

**Published:** 2023-11-03

**Authors:** Kangling Xie, Cui Li, Mingzhu Wang, Siqian Fu, Ying Cai

**Affiliations:** National Clinical Research Center for Geriatric Disorders, Department of Rehabilitation, Xiangya Hospital Central South University, Changsha, Hunan, China

**Keywords:** peripheral blood-derived exosomes, microRNA-135a-3p, ErbB, ATM, Type 2 diabetes, vascular injury, human vascular smooth muscle cells

## Abstract

**Objective:**

Diabetes pathology relies on exosomes (Exos). This study investigated how peripheral blood Exo-containing microRNAs (miRNAs) cause vascular injury in type 2 diabetes (T2D).

**Methods:**

We removed DEmiRNA from T2D chip data from the GEO database. We isolated Exo from 15 peripheral blood samples from T2D patients and 15 healthy controls and measured Exo DEmiRNA levels. We employed the intersection of Geneards and mirWALK database queries to find T2D peripheral blood mRNA-related chip target genes. Next, we created a STRING database candidate target gene interaction network map. Next, we performed GO and KEGG enrichment analysis on T2D-related potential target genes using the ClusterProfiler R package. Finally, we selected T2D vascular damage core genes and signaling pathways using GSEA and PPI analysis. Finally, we used HEK293 cells for luciferase assays, co-cultured T2D peripheral blood-derived Exo with HVSMC, and detected HVSMC movement alterations.

**Results:**

We found 12 T2D-related DEmiRNAs in GEO. T2D patient-derived peripheral blood Exo exhibited significantly up-regulated miR-135a-3p by qRT-PCR. Next, we projected miR-135a-3p’s downstream target mRNA and screened 715 DEmRNAs to create a regulatory network diagram. DEmRNAs regulated biological enzyme activity and vascular endothelial cells according to GO function and KEGG pathway analysis. ErbB signaling pathway differences stood out. PPI network study demonstrated that DEmRNA ATM genes regulate the ErbB signaling pathway. The luciferase experiment validated miR-135a-3p and ATM target-binding. Co-culture of T2D patient-derived peripheral blood Exo with HVSMC cells increases HVSMC migration, ErbB2, Bcl-2, and VEGF production, and decreases BAX and ATM. However, miR-135a-3p can reverse the production of the aforesaid functional proteins and impair HVSMC cell movement.

**Conclusion:**

T2D patient-derived peripheral blood Exo carrying miR-135a-3p enter HVSMC, possibly targeting and inhibiting ATM, activating the ErbB signaling pathway, promoting abnormal HVSMC proliferation and migration, and aggravating vascular damage.

## Introduction

Type 2 diabetes (T2D) is a prevalent endocrine disorder and remains a public health burden with increased incidence and mortality risks (1). An estimation from The International Diabetes Federation reveals that the number of people suffering from diabetes is expected to rise to 629 million by 2045, and an increased incidence of T2D has been observed in most countries (2). It is well known that the risk factors for T2D include age, lifestyle, and diet. Insulin resistance and β-cell dysfunction are the most critical pathophysiological features of type 2 diabetes (3). T2DM-related vascular manifestations can be attributed to cellular dysfunction in the vascular system during the complex response to environmental stimuli, among which endothelial and smooth muscle cells are the main participants (4). Endothelial cells are the initiating factor of vascular endothelial injury and were taken as the research object in our previous studies. Moreover, vascular smooth muscle cells (VSMC) accumulate in large numbers in atherosclerotic lesions, which is the cytopathological basis for promoting intima thickening, lipid deposition, plaque formation, and stability, and play a vital role in T2D macrovascular lesions characterized by arteriosclerosis (5). Thus, we focused on vascular injury in T2D.

Exosomes are extracellular vesicles that are commonly secreted by most cells. These vesicles contain an array of important bioactive molecules, such as lipids, carbohydrates, proteins, DNA, and RNA. As a result, they play a crucial role in facilitating cell-to-cell communication and various other biological activities (6–9). It has been previously reported that Exos can prevent T2D (10). Strikingly, Exos derived from human peripheral blood can serve as a delivery cargo system for microRNAs (miRNAs), exerting a therapeutic role in cardiac diseases (11). As previously reported, The upregulation of miR-135a could protect against myocardial ischemic/reperfusion injury in diabetic mice by diminishing TXNIP expression (12). Moreover, inhibition of miR-135a-5p was reported to diminish the proliferation of VSMCs and vascular remodeling in rats with hypertension (13). Ataxia-telangiectasia mutated (ATM) is recognized as a Ser/Thr protein kinase and executes a regulatory function on DNA damage signaling response pathways (14). Intriguingly, it was previously reported that ATM may play an essential regulatory role in insulin resistance, atherosclerosis, and metabolic syndrome (15). The epidermal growth factor receptor 2 (EGFR2) is one of the most well-studied breast cancer genes. The ebb-2 signaling pathway can mediate various physiological responses, including cellular proliferation and migration. It is also instrumental in promoting wound healing and vascular smooth muscle proliferation in diabetic patients. An abnormally low level of ATM was discovered in epidermal growth factor receptor 2 (ErbB2)-triple-negative breast cancer, suggesting the possible regulatory relationship between ATM and ErbB2.

Furthermore, the ErbB signaling pathway can mediate various physiological responses, including cell proliferation and motility (16). It has been revealed that repression of EGFR could prevent obesity-induced vascular dysfunction (17). Considering the findings above, we propose a hypothesis in this current study that Exos-containing miR-135a-3p derived from the peripheral blood can regulate vascular injury in T2D with the involvement of the ErbB/ATM axis.

## Methods

### Ethical statement

This study was conducted with the approval of the Medical Ethics Committee, Xiangya Hospital, Central South University(202305356).All patients signed the informed consent form before the experiment.

### Gene expression omnibus microarray data acquisition and analysis

This article uses the Gene Expression Omnibus (GEO) database (https://www.ncbi.nlm.nih.gov/gds) to obtain three T2D expression profile datasets, namely GSE44093, GSE26168, and GSE70318. Differential miRNA analysis data were from 10 peripheral blood samples of T2D patients and 10 normal control peripheral blood samples in the GSE44093 dataset, 7 plasma samples from T2D patients and 10 normal control peripheral blood samples in the GSE26168 dataset, and 8 plasma samples from T2D patients and 8 normal control peripheral blood samples in the GSE70318 dataset. Differential mRNA analysis data were obtained from 8 T2D patient plasma samples and 8 normal control peripheral blood samples in the GSE26168 dataset.

Differential analysis was performed using the “limma” package in R language (https://www.ncbi.nlm.nih.gov/gds), with |logFC|>1 and significant P<0.05 as the selection criteria for differential expression of mRNA and miRNA. Subsequently, the volcano plot was plotted using the “ggplot2” package in R, and the heatmap of differential gene expression was drawn using the “heatmap” package in R.

### Intersection gene selection

The Venn diagram tool (http://bioinformatics.psb.ugent.be/webtools/Venn/) was used to perform intersection operations on the high-expression differential miRNAs in T2D patient peripheral blood samples in the GSE44093, GSE26168, and GSE70318 chips, to obtain candidate DEmiRNAs.

### DEmiRNA downstream regulation mRNA prediction

The miRDB (http://mirdb.org/) and Norwalk (http://mirwalk.umm.uni-heidelberg.de/) databases were used to predict downstream mRNAs of target genes. In addition, the Draw Venn Diagram tool was used again to perform intersection operations on T2D-related mRNA expression profile data from the GSE26168 differential analysis results to further determine the downstream regulation mRNA of the target miRNA in T2D. Finally, the miRNA-mRNA targeting network was constructed using the Cytoscape (v3.6.0) visualization software.

### Functional enrichment analysis

The “ClusterProfiler” software package in R was used to perform functional enrichment analysis on the filtered downstream regulation mRNAs of the target miRNA. Fisher’s test was used to determine significantly enriched GO and KEGG pathways, with P<0.05 considered statistically significant. In addition, gene set enrichment analysis (GSEA) (http://software.broadinstitute.org/gsea/) based on the gene expression profile of T2D mRNA in the GSE26168 dataset was used to investigate the pathway information enriched by the downstream regulation mRNAs of the target miRNA.

### Screening angiogenesis-related genes from the GeneCards and STRING databases

The top 100 differentially expressed (DEmRNAs) with |logFC| values from the GSE26168 dataset were selected. These DEmRNAs and the ErbB pathway-related genes were inputted into the STRING database (https://string-db.org/) to obtain a protein-protein interaction (PPI) network. The co-expression relationship between key DEmRNAs in vascular tissue and ErbB pathway-related genes was verified using the ChIPBase v3.0 website (https://rnasysu.com/chipbase3/index.php). The “Angiogenesis”-related genes were retrieved from the GeneCards database (https://www.genecards.org/) and sorted in descending order based on the Relevance score, resulting in the selection of the top 100 angiogenesis-related genes. To analyze the correlation of genes associated with type 2 diabetes (T2D), the GENEMANIA website (http://genemania.org/) was utilized. The identified T2D-related genes and the selected T2D-associated genes were imported into the STRING database (https://string-db.org/) for protein-protein interaction analysis. The analysis was limited to the human species, and the resulting network regulatory relationships were visualized using Cytoscape software (v3.6.0).

### Isolation and identification of exosomes from peripheral blood

We collected peripheral blood samples from 15 T2D patients and 15 healthy volunteers, and all qualified peripheral blood samples were coagulated at room temperature. Then, the serum samples were centrifuged at 3000 g and 4°C for 10 minutes, followed by centrifugation at 10000 g and 4°C for 30 minutes to remove cell debris. Next, the supernatant was ultracentrifuged at 100000 g and 4°C for 2 hours to precipitate particles. Next, the particles were washed with PBS and filtered through a 0.22 μm filter. Finally, the particles were ultracentrifuged again at 100000 g and 4°C for 2 hours to precipitate Exosomes. These Exosomes particles were suspended in 100μL PBS.

Exos were identified under a transmission electron microscope. In short, the samples were adsorbed on a carbon-coated nickel mesh and stained with 2% methylamino tungstate for 5 minutes. The stain was subsequently wiped off the mesh with filter paper, followed by two washes with distilled water drops. After treatment, the dried samples were examined with a JEM-1230 electron microscope (Nihon Denshi, Tokyo, Japan) at an accelerated voltage of 80 kV (18). Finally, the particle size was analyzed by nanoparticle tracking analysis (NS300, Malvern Instruments Ltd., Malvern, UK) (19).

The western blot assay identified Exos surface markers. After the suspension was concentrated, the protein content was determined using BCA kits (23227, Thermo Fisher Scientific Inc., Waltham, MA, USA). The sodium dodecyl sulfate-polyacrylamide gel electrophoresis (SDS-PAGE) gel was prepared, and protein denaturation and electrophoresis were performed. Finally, the expression of the Exo-specific markers, namely, protein tumor susceptibility gene 101 (TSG101), CD63, and CD81 (20), were determined, respectively.

### Human vascular smooth muscle cell culture and transfection

Human vascular smooth muscle cells HVSMC (Bio-73393, Beijing Beiao Bowei Biological Technology Co., Ltd., Beijing, China) were cultured using Ham’s F-12K medium (PM150910, Beijing Beiao Bowei Biological Technology Co., Ltd., Beijing, China) containing 0.05mg/ml VitC + 0.01mg/ml Insulin + 0.01mg/ml Transferrin + 10 ng/ml Sodium Selenite + 0.03mg/ml ECGs + 10% FBS + 10mM HEPES + 10mM TES + 1% P/S. The cells were cultured in a cell culture incubator at 37°C, 5% CO2, and saturated humidity. Trypsin was used for digestion and subculture when the cell growth fusion degree reached over 90%. We used inhibitor-NC and miR-23a inhibitors in the cell groups and transfected them with Lipo3000 (Sigma Co., USA). When cells were in the logarithmic growth phase, and the fusion rate reached 30%, 2×106 TU of a corresponding slow virus was added into 1mL of serum-free and non-antibiotic-containing medium with 5µg Poly-brene, mixed well, soaked for 48 hours, and then 1μg/mL puromycin was added into each well to screen transfected cells.

### Extracellular vesicle uptake experiment

Isolated Exos were incubated with red fluorescent dye PKH26 (Sigma-Aldrich, USA) at room temperature for 5 minutes. The Exos were washed with PBS, centrifuged at 100,000 g for 90 minutes, suspended in a basic culture medium, and then incubated with HVSMC cells at 37°C for 12 hours. The cells were fixed with 4% paraformaldehyde, and their nuclei were stained with 10 μg/mL Hoechst 33342 (C1025, Beyotime, Nantong, China) for 10 minutes. Exos uptake was observed using a confocal laser scanning microscope (Zeiss LSM710, Germany) with an excitation wavelength of 350 nm and 551 nm.

### Co-culture of HVSMCs and peripheral blood-derived Exos

After co-cultivating 20 μL of fluorescence-labeled Exo (at a concentration of 20 μg/mL) with HVSMC cells seeded in a 24-well plate, exhibiting a fusion degree of 50-60%, the experiment proceeded for 48 hours (21). The co-culture was maintained for 48 hours before further experimentation. The co-culture groups included hC-exo (human-control-exo co-cultured with normal peripheral blood-derived Exos and HVSMC cells), hC-exo + miR-135a-3p mimic (human-control-exo co-cultured with HVSMC cells transfected with miR-135a-3p mimic and normal peripheral blood-derived Exos), hT2D-exo (human-T2D-exo co-cultured with HVSMC cells derived from diabetic patients), hT2D-exo + inhibitor-NC (hT2D-exo co-cultured with HVSMC cells transfected with inhibitor-NC and derived from diabetic patients), hT2D-exo + miR-135a-3p inhibitor (hT2D-exo co-cultured with HVSMC cells transfected with miR-135a-3p mimic and derived from diabetic patients), and hT2D-exo + miR-135a-3p inhibitor+AZD0156 (hT2D-exo co-cultured with HVSMC cells transfected with miR-135a-3p mimic, derived from diabetic patients, and treated with 100 nm AZD0156).

### Dual-luciferase gene reporter assay

The dual-luciferase experiment was used to verify the targeted relationship between miR-135a-3p and ATM. The ATM 3’ UTR binding site to miR-135a-3p was identified through the miRWALK database, and the PCR product of the ATM gene 3’UTR region was cloned into the pmirGLO (Promega Co., Ltd., USA, E1330) downstream of the Luciferase gene, and named pATM-WT. Point mutations were made to disrupt the predicted binding site between miR-135a-3p and the target gene, and the resulting vector was named pATM-Mut. The pRL-TK vector (Promega Co., Ltd., USA, E2241) expressing Renilla luciferase was used as an internal reference. HEK293 cells (ATCC, USA, CRL-11268) were transfected with miR-135a-3p mimic and negative control, respectively, and were harvested using the Dual-Luciferase^®^ Reporter Assay System (Promega, USA, E1910) 24 hours later for cell lysis and collection of supernatants. The activity of the Firefly luciferase reporter gene was detected using the Tecan Infinite 200 (Tecan Group Ltd., Germany, Crailsheim), while the activity of the Renilla luciferase reporter gene was detected after the addition of the stop buffer. The ratio of Firefly luciferase to Renilla luciferase (FL/RL) was used as the relative luciferase activity. The experiment was repeated 3 times.

### Transwell assay

A 24-well Transwell cell culture chamber with an 8.0 μm porous polycarbonate filter was used to evaluate the migration of HVSMC cells in different groups. Before migration determination, all cells were starved for 12 hours in serum-free media. Next, HVSMC cells were digested with trypsin, suspended in 0.2% BSA at a concentration of 4×105/mL, and then 200 μL of 0.2% BSA was added to the filter. The cells were incubated for 48 hours at 37°C with 5% CO2, after which the insert was removed, and the filter was washed with 0.1mol/L phosphate-buffered saline. The migrated cells were fixed, stained with hematoxylin, and checked under a microscope. The fluorescence intensity of the migrated cells was measured at 570 nm using an enzyme-linked immunosorbent assay.

### Reverse transcription-quantitative polymerase chain reaction

Total RNA was extracted from peripheral blood-derived exosomes of healthy controls and T2D patients using Trizol reagent (Invitrogen, USA) and reverse transcribed into cDNA using TaqMan MicroRNA Assays Reverse Transcription Primer (Applied Biosystems, USA, Cat. No. 4427975) according to the manufacturer’s instructions. The conditions for the reverse transcription reaction were: 37°C for 30 min, 85°C for 5 s. Five microliters of the above cDNA product were used as a template for PCR amplification. The PCR reaction system consisted of 25 μL: 5 μL of cDNA product, 13 μL of 2 × QuantiTect SYBR Green RT-PCR Master Mix, 0.5 μL of upstream and downstream primers (10 μmol/μL each), and 6 μL of nuclease-free water. The reaction conditions were: 95°C for 5 min, followed by 45 cycles of 95°C for 20 s → 60°C for 1 min → 72°C for 30 s. The relative differential expression of the target genes was calculated using the 2-ΔΔCt method, where 2-ΔCt = Ct (target gene) - Ct (reference gene) and 2-ΔΔCt = Ct (experimental) - Ct (control). Primer sequences are listed in **Table S1**.

### Western blot

Total protein was extracted from cells using RIPA lysis buffer (Beyotime, Shanghai, China) containing PMSF and incubated on ice for 30 min at 4°C, followed by centrifugation at 8000g for 10 min. The total protein concentration was determined using a BCA protein assay kit (BCA1 and B9643, Sigma, USA). Fifty micrograms of protein were dissolved in 2 × SDS loading buffer and boiled for 5 min at 100°C. The sample was then separated by SDS-PAGE and transferred onto a PVDF membrane. At room temperature, the PVDF membrane was blocked with 5% skimmed milk powder for 1 h. The membrane was then incubated with diluted antibodies [ATM (ab32420, 1:1000, Abcam), BAX (ab32503, 1:1000, Abcam), ErbB2 (ab237715, 1:1000, Abcam), Bcl-2 (ab32124, 1:1000, Abcam), VEGF (ab52917, 1:10000, Abcam), GAPDH (ab181602, 1:10000, Abcam)] overnight at 4°C. After washing with TBST three times, the membrane was incubated with goat anti-rabbit HRP-conjugated secondary antibody (ab205718, 1:2000, Abcam) for 1 h at room temperature. Next, the membrane was washed with TBST and placed onto a clean glass plate. Finally, the membrane was developed using an ECL chemiluminescence kit (BB-3501, Amersham, UK) by mixing equal amounts of A and B reagents in the darkroom and placing the mixture on the membrane. The signal was detected using the Bio-Rad imaging system (Bio-Rad, USA) and analyzed using Quantity One v4.6.2 software. The protein content was calculated by the ratio of the grayscale value of the corresponding protein band to that of the GAPDH protein band, and three independent experiments were performed.

### Statistical analysis

Statistical analysis was performed using SPSS version 21.0 (IBM SPSS Statistics, Chicago, IL, USA). Data are presented as mean ± SD. For normally distributed and homogenous data between two groups, the unpaired t-test was used. One-way ANOVA was used to compare multiple groups, followed by Tukey’s *post hoc* test. Repeated measures ANOVA was used to compare multiple time points, followed by Tukey’s *post hoc* test. A P value less than 0.05 was considered statistically significant.

## Results

### Bioinformatics screening identified 12 differentially expressed miRNAs associated with T2D

**Figure 1:**
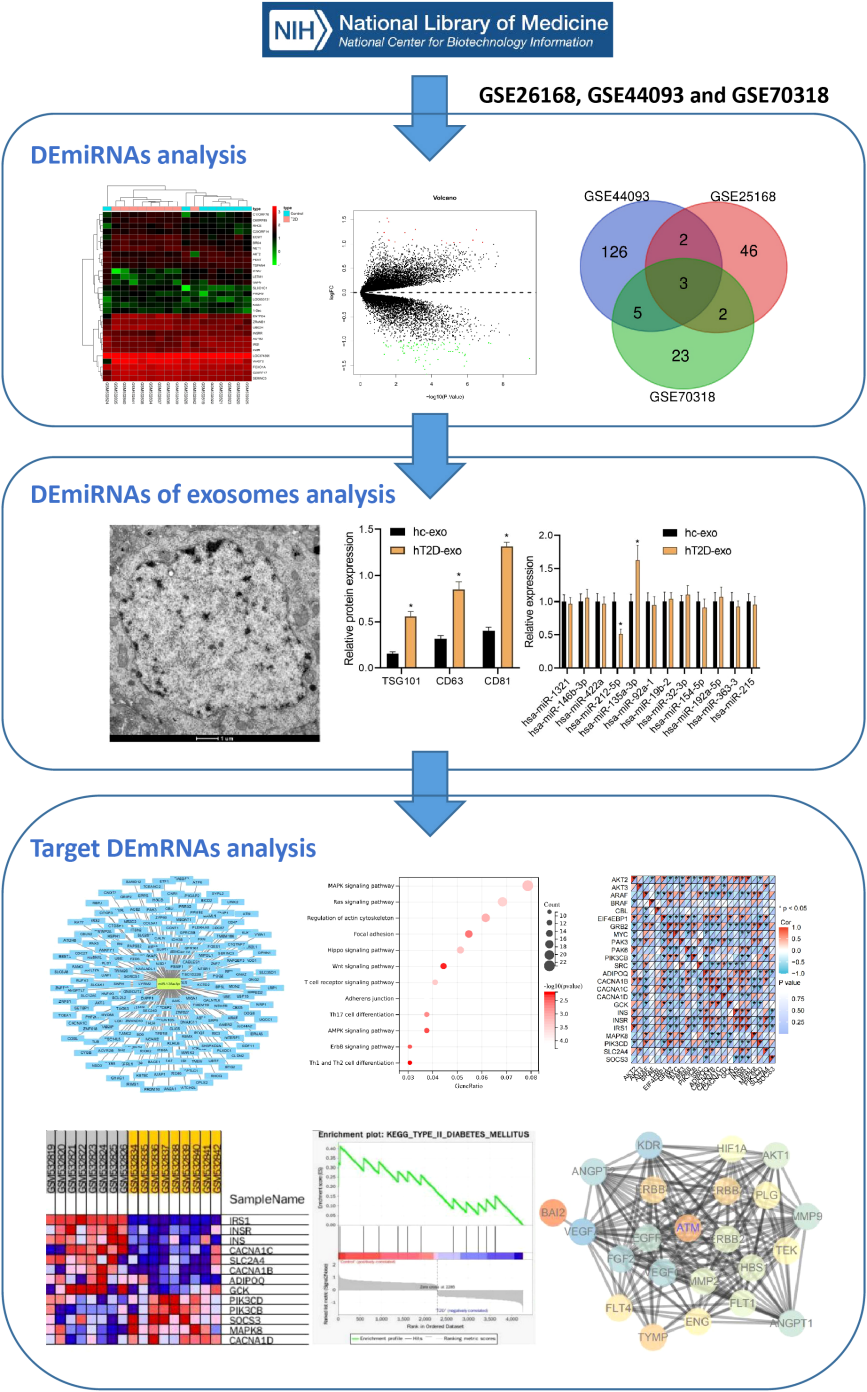
The bioinformatics screening flow chart of key genes involved in vascular injury in T2D.

T2D is a common metabolic disease with an increasing incidence over time that can cause various cardiovascular complications, posing a serious threat to human health. Our study aimed to screen key miRNAs related to T2D-induced vascular injury and investigate their expression levels in Exosomes derived from peripheral blood. We also constructed miRNA-mediated signaling pathways through online databases to further study the biological mechanisms by which Exosomes carrying miRNAs from peripheral blood act in the pathogenesis of T2D, providing a theoretical basis for a deeper understanding of the mechanisms of T2D vascular injury and potential diagnostic and therapeutic targets. The bioinformatics analysis flow is illustrated in **Figure 1**.

**Figure 2:**
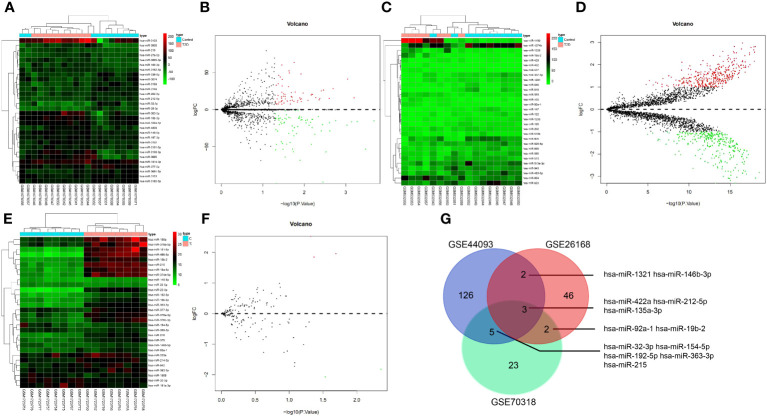
The miRNA screening related to T2D vascular injury. **(A, B)** heat map and volcano plot of differentially expressed miRNAs in T2D samples and normal samples in GSE26168 chip; **(C, D)** heat map and volcano plot of differentially expressed miRNAs in T2D samples and normal samples in GSE44093 chip; **(E, F)** heat map and volcano plot of differentially expressed miRNAs in T2D samples and normal samples in GSE70318 chip. In the volcano plot, green circles represent downregulation, red circles represent upregulation, and gray circles represent no significant differences. The horizontal axis represents the logarithmic value of the fold change (FC) between different groups, with 2 as the base, i.e., log2(FC); the vertical axis represents the negative logarithmic value of the significance test P-value, with 10 as the base, i.e., -log10 (p-value). **(G)** Venn processing of DEmiRNA in GSE26168, GSE44093, and GSE70318 datasets.

First, 53 DEmiRNAs were obtained from the GSE26168 dataset, of which 17 were up-regulated, and 36 were downregulated (**Figures 2A, B**). 136 DEmiRNAs were identified from the GSE44093 dataset, of which 64 were up-regulated and 72 were downregulated (**Figures 2C, D**). Furthermore, 33 DEmiRNAs were identified from the GSE70318 dataset, including 15 up-regulated DEmiRNAs and 18 downregulated ones (**Figures 2E, F**). Finally, 12 DEmiRNAs (hsa-miR-1321, hsa-miR-146b-3p, hsa-miR-422a, hsa-miR-212-5p, hsa-miR-135a-3p, hsa-miR-92a-1, hsa-miR-19b-2, hsa-miR-32-3p, hsa-miR-154-5p, hsa-miR-192-5p, hsa-miR-363-3, and hsa-miR-215) were obtained by intersecting the results from the GSE26168, GSE44093 and GSE70318 datasets (more than three times in overlapping) (**Figure 2G**).

### Isolation and characterization of peripheral blood-derived Exos

**Figure 3:**
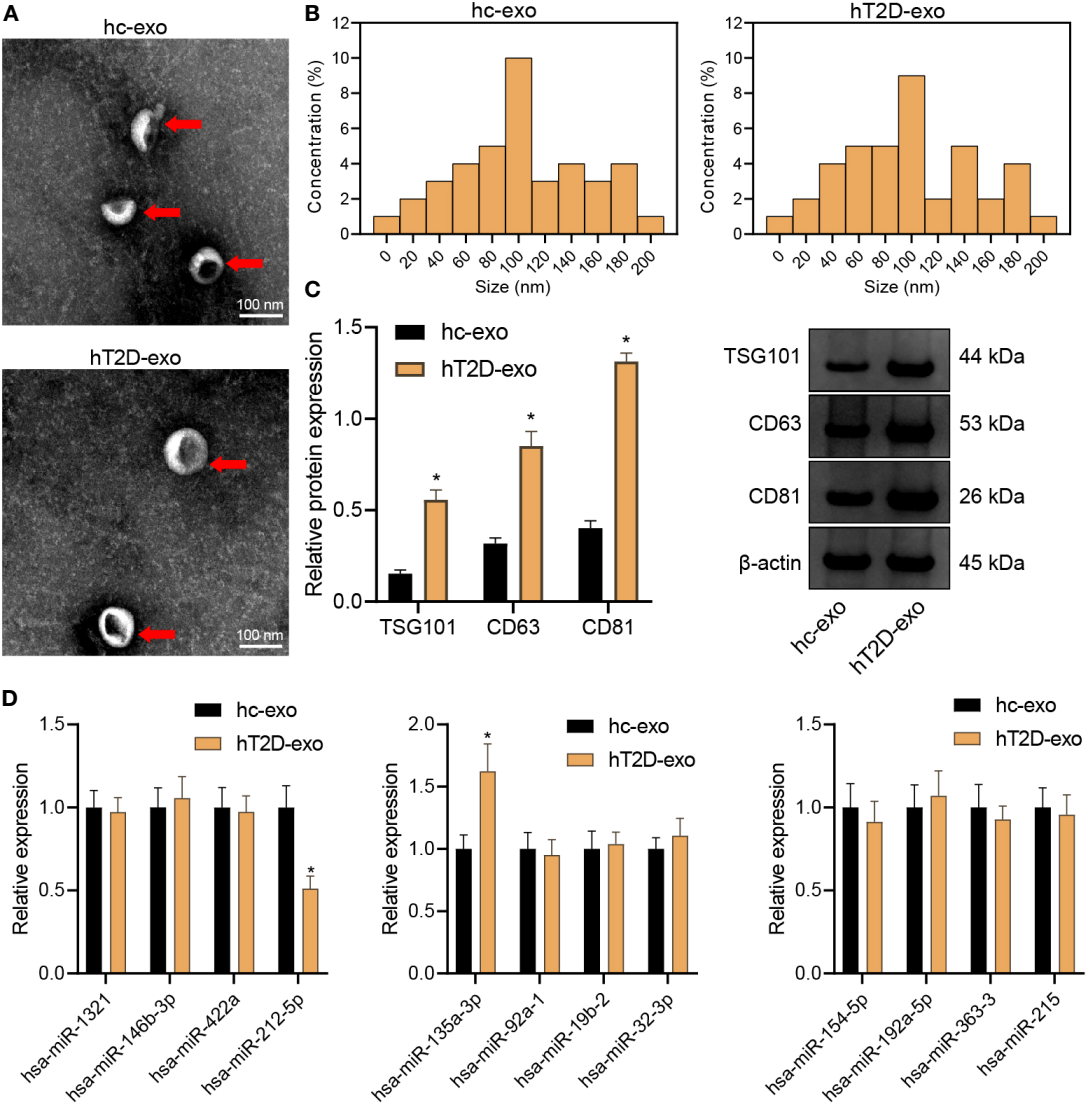
Isolation and characterization of peripheral blood-derived Exos. **(A)** The morphology of peripheral blood-derived Exos was observed under a transmission electron microscope (scan bar = 100 nm). **(B)** Nanoparticle tracking analysis was used to assess the size distribution of the Exos. **(C)** Western blot assay was used to determine the expression of Exo marker proteins TSG101, CD63, and CD81. **(D)** RT-qPCR was used to determine the expression of DEmiRs in peripheral blood-derived Exos. *p < 0.05 *vs.* hC-Exos.

Subsequently, peripheral blood-derived Exos were isolated and identified with a transmission electron microscope. The Exos showed a solid, dense body with a typical double-layer membrane structure, either disk- or cup-shaped, with an average diameter of about 90 nm (**Figures 3A, B**). In addition, compared with hC-Exos, Exo marker proteins TSG101, CD63, and CD81 were more abundant in hT2DM-Exos (**Figure 3C**). The results suggested that both hC-Exos and hT2D-Exos were successfully extracted, and the Exos had more content in the peripheral blood of T2D patients. Further detection of the expression of DEmiRNAs in peripheral blood-derived Exos showed that the expression of miR-135a-3p in hT2D-Exos was significantly higher than that in hC-Exos (**Figure 3D**). Therefore, miR-135a-3p was selected as the target gene for further analyses.

### Screening of downstream target mRNAs of miR-135a-3p

**Figure 4:**
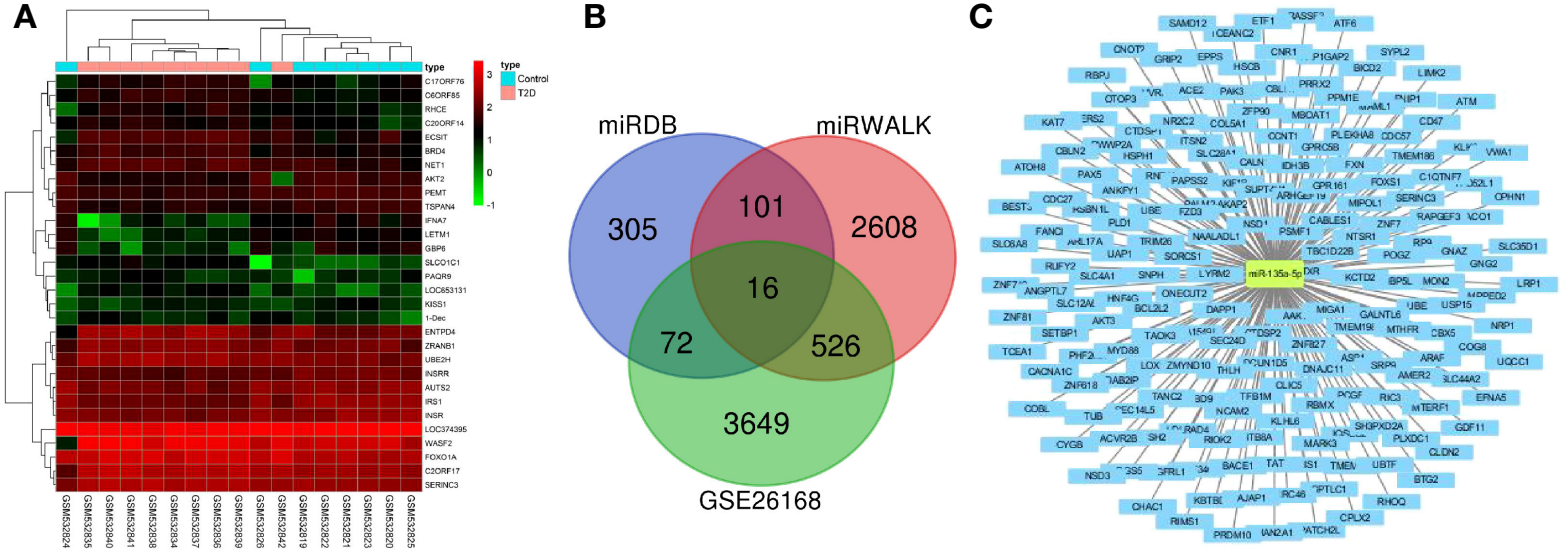
Screening of downstream mRNAs of miR-135a-3p. **(A)** The mRNA heatmap for T2D and normal samples in the GSE26168 dataset. **(B)** The Venn map for screening results from the GSE26168 dataset, DEmRNA, miEDB, and miRWALK websites. **(C)** The network of miR-135a-3p regulatory mRNAs was drawn using the Cytoscape software.

4263 DEmRNAs were retrieved from the GSE26168 dataset, among which 825 were up-regulated, and 3438 were downregulated (**Figure 4A**). Furthermore, the miRDB and miRWALK websites screened 494 and 3251 downstream regulatory mRNAs of miR-135a-3p, respectively. Next, 715 DEmRNAs were screened by intersecting the above three data groups using Venn analysis (**Figure 4B**). Afterward, the network of miR-135a-3p regulatory mRNAs was constructed using the Cytoscape software (**Figure 4C**), and finally, the DEmRNAs were acquired for subsequent analyses.

### The downstream DEmRNAs of miR-135a-3p were mainly involved in the regulation of enzyme activity and vascular endothelial cells

**Figure 5:**
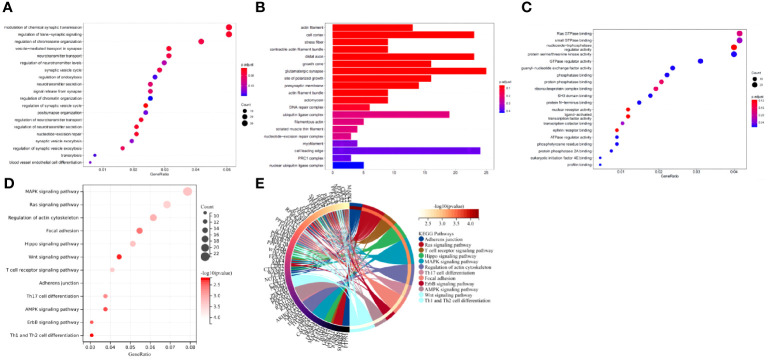
The downstream target DEmRNAs of miR-135a-3p were mainly involved in regulating enzyme activity and vascular endothelial cells. **(A)** GO functional analysis of DEmRNAs at the biological process level. **(B)** GO functional analysis of DEmRNAs at the cellular component level. **(C)** GO functional analysis of DEmRNAs at the molecular function level. **(D)** KEGG pathway enrichment analysis of DEmRNAs. The dot size represents the number of selected genes, and the color represents the *p*-value of enrichment analysis. **(E)** In the circle diagram of KEGG enrichment results, the right side of the outermost circle is Term, the left side is the color corresponding to the gene, and the left inner circle represents the *p*-value of the corresponding pathway.

GO and KEGG pathway analyses of the DEmRNAs in the peripheral blood samples of T2D patients were performed. The results from GO functional analysis demonstrated that DEmRNAs were mainly enriched in items such as regulation of GTPase activity, regulation of cell morphogenesis, regulation of chromosome organization, positive regulation of GTPase activity, valuable chromatin modification, and blood vessel endothelial cell differentiation (**Figure 5A**). In cell component, the DEmRNAs were mainly enriched in items such as glutamate synapse, cell leading edge, cell cortex, distal axon, membrane region, growth cone, site of polarized growth, presynaptic membrane, action fiber, and stress fiber (**Figure 5B**). In the molecular function, the DEmRNAs were mainly enriched in entries such as small GTPase binding, nucleoside-triphosphatase regulator activity, protein serine/threonine kinase activity, actin binding, ubiquitin-protein transferase activity, GTPase regulator activity, DNA-binding transcription factor binding, active transmembrane transporter activity, GTPase activator activity and cadherin binding (**Figure 5C**). Overall, the downstream DEmRNAs of miR-135a-3p were primarily associated with the regulation of biological enzymatic activity, cell morphology, differentiation of vascular endothelial cells and were enriched in the cell cortex and stress fibers. Moreover, the molecular function of DEmRNAs mainly participated in the modulation of cell-related enzyme activity and receptor activity.

KEGG pathway analysis revealed that DEmRNA were mainly enriched in items such as the MAPK signaling pathway, calcium signaling pathway, signaling pathways regulating proliferation of stem cells, Rap1 signaling pathway, chemokine signaling pathway, ErbB signaling pathway, and FOXO signaling pathway (**Figures 5D, E**). Generally speaking, the downstream DEmRNAs of miR-135a-3p regulated multiple signaling pathways.

### ATM was involved in vascular injury in T2D through the ErbB signaling pathway

**Figure 6:**
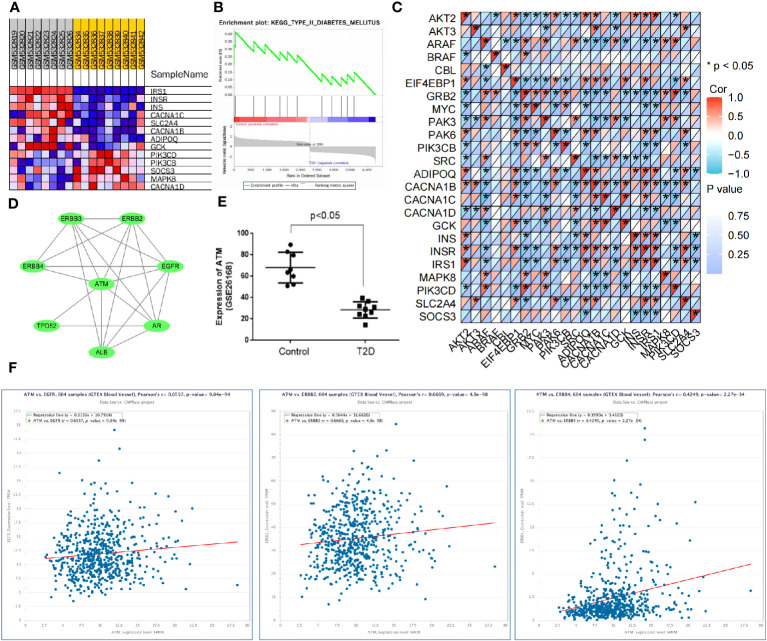
ATM is involved in vascular injury in T2D through the ErbB signaling pathway. **(A, B)** GSEA was used to screen the genes related to T2D occurrence in the GSE26168 dataset. **(C)** The matrix correlation calculation tool was used to analyze the correlation between genes regulating the ErbB signaling pathway and T2D-related genes. **(D)** The PPI network for the regulation of ATM on the ErbB signaling pathway. **(E)** The expression of ATM in the GSE26168 dataset. **(F)** The ChIPBase website validated the co-expression relationship between ATM and the ErbB1, ErbB2, and ErbB4 genes in vascular tissues.

Combined with the results of the KEGG pathway analysis, the top five signaling pathways in terms of *p*-value were screened for literature analysis. Among them, the ErbB signaling pathway participated in the occurrence and development of T2D by mediating the systemic insulin level (22). It affected the angiogenic mechanism by regulating the activity of various factors in the pathway (23). Based on the KEGG pathway analysis results, the DEmRNAs involved in modulating the ErbB signaling pathway included GRB2, PIK3CB, MYC, AKT2, BRAF EIF4EBP1, SRC, PAK6, CBL, PAK3, ARAF, and AKT3. Meanwhile, GSEA was conducted to explore the relationship between molecular function and gene expression in the GSE26168 dataset and genes related to T2D (IRS1, INSR, INS, CACNA1C, SLC2A4, CACNA1B, ADIPOQ, GCK, PIK3CD, PIK3CB, SOCS3, MAPK8, and CACNA1D) were screened (**Figures 6A, B**). The correlation between genes involved in mediating the ErbB signaling pathway and T2D-related genes was analyzed using the matrix correlation calculation tool (**Figure 6C**). The ErbB signaling pathway may be involved in the occurrence and development of T2D.

The relationship between the DEmRNAs and ErbB signaling pathway was further analyzed based on the above findings. The top 100 DEmRNAs in terms of *p*-value and the genes related to the ErbB signaling pathway (RNF41, AAK1, RNF41, AAK1, EBB3, and ErbB4) were introduced into the STRING database. With the species limited to “humans”, a PPI relationship was established. According to previous literature, ATM regulated diabetes mellitus (15). The data regarding the regulation of the ErbB signaling pathway by ATM were input into the Cytoscape software to construct a PPI network (**Figure 6D**). ATM was found to be significantly downregulated in the peripheral blood samples of T2D patients in the GSE26168 dataset (**Figure 6E**). Additionally, ChIPBase v3.0 revealed a significant positive correlation between ATM and the expression of ErbB1, ErbB2, and ErbB4 in vascular tissues (**Figure 6F**). Overall, these results suggest that ATM may be involved in the occurrence of T2D vascular damage by regulating the ErbB signaling pathway.

### ATM and ErbB signaling pathways might participate in vascular injury in T2D

**Figure 7:**
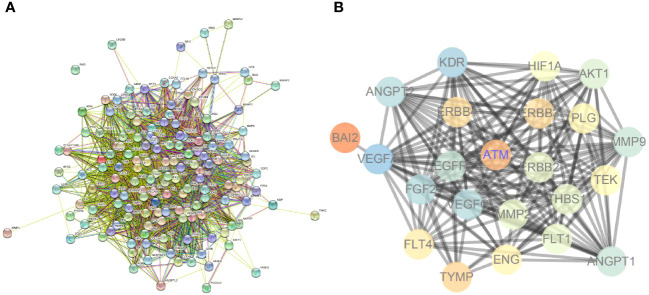
ATM and ErbB signaling pathways may participate in vascular injury in T2D. **(A)** The PPI network for angiogenesis-related genes. **(B)** PPI network for regulating ATM and ErbB signaling pathways on angiogenesis.

In addition, angiogenesis-related genes were screened through the GeneCards database. A PPI network of these genes was constructed using the STRING database (**Figure 7A**). The ATM and ErbB signaling pathway-related genes, the core genes of ATM, ErbB signaling pathway-related and angiogenesis-related genes were further introduced into the STRING database to obtain the PPI relationship (the species was limited to “human”). Next, the data were imported into the Cytoscape software to establish the PPI network (**Figure 7B**), which involved 25 nodes and 177 edges (PPI enrichment *p*-value < 1.0e-16). These results implied that ATM and ErbB signaling pathways may be involved in the occurrence of vascular injury in T2D.

### Peripheral blood-derived Exos from T2D patients carrying miR-135a-3p aggravate vascular injury in T2D through regulation of the ATM/ErbB axis

Peripheral blood-derived Exos play a pivotal role in the pathophysiology of T2D. These Exos from T2D patients have been found to harbor miR-135a-3p. Our recent investigations suggest that this specific microRNA plays a significant role in mediating vascular injury, a common complication in T2D patients.

**Figure 8:**
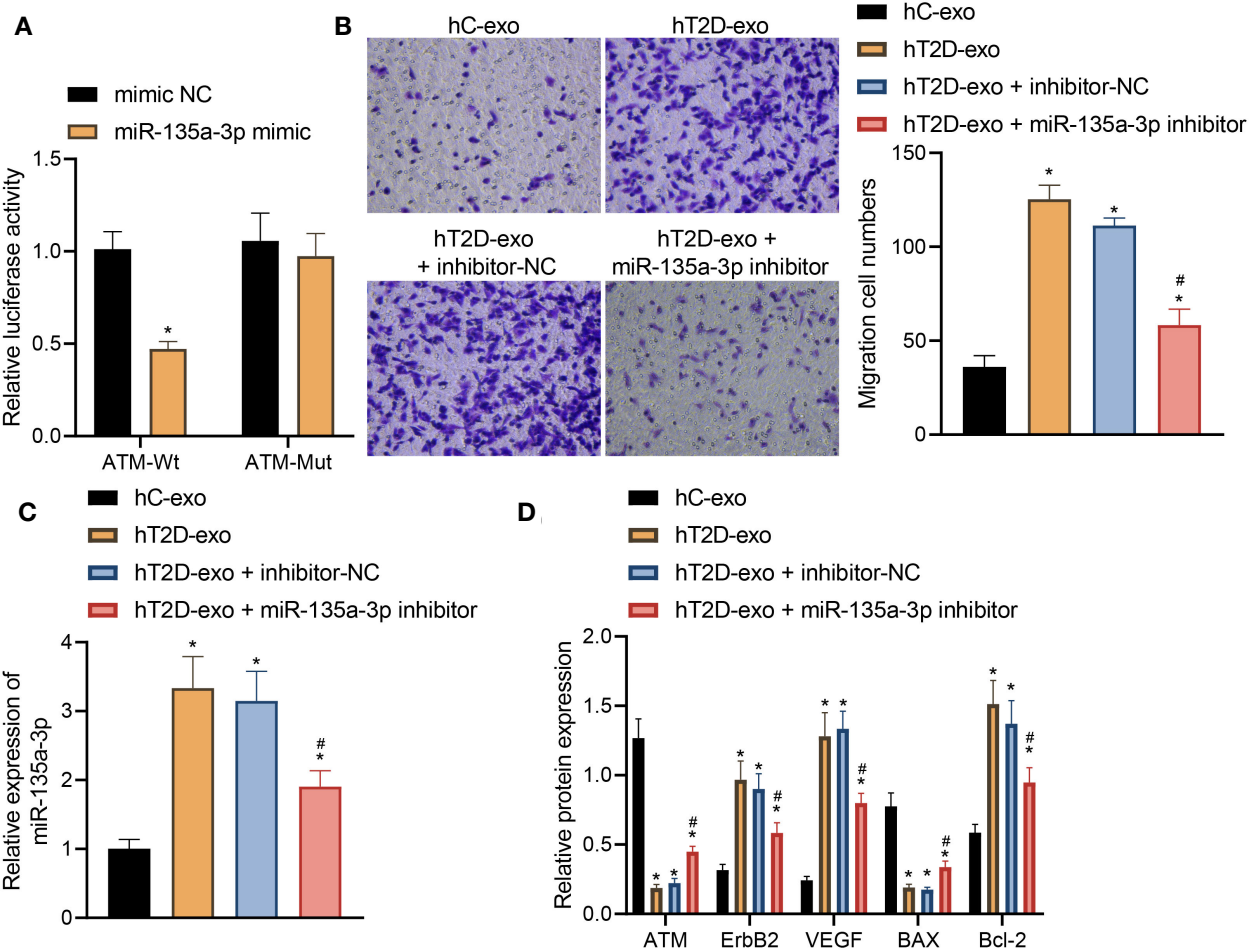
Peripheral blood-derived Exos from T2D patients carrying miR-135a-3p alleviate vascular injury in T2D by regulating the ATM/ErbB axis. **(A)** Dual-luciferase gene reporter assay verified the targeting relationship between miR-135a-3p and ATM. **(B)** The transwell assay was used to detect the migration ability of HVSMCs. **(C)** The expression of miR-135a-3p in HVSMCs was determined by RT-qPCR. **(D)** The expression of ATM, ErbB2, Bcl-2, VEGF, and Bax was determined by Western blot. *p < 0.05 *vs.* hC-Exos. #*p* < 0.05 *vs.* hT2D-Exos.

A dual-luciferase gene reporter assay was utilized to confirm the targeting relationship between miR-135a-3p and ATM (**Figure 8A**). Notably, this relationship is of immense significance given the vital role of ATM in cellular processes. Our results revealed a distinct trend: compared to healthy control-derived Exos (hC-Exos), the T2D patient-derived Exos (hT2D-Exos) or hT2D-Exos treated with a non-specific inhibitor (inhibitor NC) significantly enhanced the migration capabilities of human vascular smooth muscle cells (HVSMCs). However, this enhanced migration was notably reduced when hT2D-Exos were treated with a miR-135a-3p inhibitor (**Figure 8B**). This indicates that the migration ability of HVSMCs is directly influenced by the expression of miR-135a-3p (**Figure 8C**).

Further insights were gained through Elisa analysis. When compared to hC-Exos, both hT2D-Exos and hT2D-Exos with inhibitor NC led to a marked decrease in the expression levels of ATM and Bax. Conversely, there was a pronounced increase in the expression levels of ErbB2, Bcl-2, and VEGF. Notably, when HVSMCs were treated with the miR-135a-3p inhibitor, these expression trends were reversed (**Figure 8D**).

In light of these findings, it can be inferred that peripheral blood-derived exosomes in T2D patients transport miR-135a-3p into HVSMCs. This, in turn, appears to downregulate ATM expression and activate the ErbB signaling pathway. The end result is an enhancement in the migratory capabilities of HVSMCs, thereby exacerbating vascular injury in T2D.

## Discussion

T2D presents a global health problem contributing to microvascular and macrovascular complications (24). Herein, we set out to explore the effects of miR-135a-3p-containing Exos derived from peripheral blood on vascular injury in T2D in combination with bioinformatics analysis and *in vitro* cell experiments and observed that they could induce the occurrence of the disease by mediating the ErbB/ATM axis.

Through bioinformatics analysis, 12 T2D-related DEmiRs were identified, and the following experiments screened hsa-miR-135a-3p as a DEmiR in the vascular injury of T2D and discovered that miR-135a-3p was up-regulated in Exos derived from the peripheral blood of T2D patients. It was reported that elevated miR-135a expression could reduce the expression of TXNIP in diabetic mice to protect against myocardial ischemic/reperfusion injury (12). The regulated miR-135a-5p/XBP1 axis by knockdown of the long non-coding RNA LINC00299 could suppress oxidized low-density lipoprotein-induced T/G human aortic VSMC injury in atherosclerosis (25). Additionally, the down-regulation of miR-135a-5p could suppress the proliferation of VSMCs and vascular remodeling in a rat model of hypertension (13). As previously reported, exosomes derived from human mesenchymal stem cells could alleviate T2D by reversing peripheral insulin resistance and ameliorating the destruction of β-cells (26). It is worthwhile pointing out that exosomes have been described as novel regulators for diabetes by delivering their non-coding RNA cargo, such as miRs (27). It was unveiled that miR135a-5p-carrying extracellular vesicles could enhance the proliferation of VSMCs in hypertensive rats by targeting FNDC5 (28). This study demonstrated that peripheral blood-derived Exos could deliver miR-135a-3p into HVSMCs and affect vascular injury in T2D.

Moreover, the experimental results indicated that the expression of hsa-miR-212-5p was significantly reduced in T2D exosomes. It is reported that miR-212-5p and its target PAFAH1B2 can inhibit vascular proliferation and contraction via the downregulation of RhoA. Therefore, the biological function of hsa-miR-212-5p for inhibiting vascular proliferation and contraction is undermined, which may aggravate vascular injury and increase the expression of miR-135a-5p.

Previous studies have shown that the high expression of miR-212-5p can downregulate RhoA through its target PAFAH1B2 to inhibit vascular proliferation and contraction (29). Our study revealed a significant decrease in the expression level of hsa-miR-212-5p in exosomes from T2D patients. Therefore, the biological function of hsa-miR-212-5p in inhibiting vascular proliferation and contraction is weakened. Additionally, the literature suggests that hsa-miR-212-5p is associated with β-cell function in diabetes (30). Overexpression of hsa-miR-212-5p significantly enhances glucose-stimulated insulin secretion in rat insulinoma cells and restores insulin responsiveness (31). Hence, the decrease in hsa-miR-212-5p expression also weakens this function. There is relatively limited literature on the association of hsa-miR-212-5p with diabetes. In future studies, more detailed and rigorous experiments can be conducted to further explore the role of hsa-miR-212-5p in the progression of type 2 diabetes.

Furthermore, by analyzing the downstream DEmRNA of miR-135a-3p, we found that the downstream DEmRNA of miR-135a-3p was mainly involved in regulating biological enzyme activity, cell morphology, and differentiation of vascular endothelial cells. Following this, KEGG and GSEA demonstrated the ErbB signaling pathway’s crucial role in T2D. Moreover, with the combination of bioinformatics analysis and literature review, the critical roles of ATM and the ErbB signaling pathway in the occurrence of T2D were highlighted and subsequently validated by *in vitro* assays. The results displayed that peripheral blood-derived Exos from T2D patients carry miR-135a-3p into HVSMCs, which activates the ErbB signaling pathway by inhibiting ATM, thereby enhancing the migration ability of HVSMCs. Notably, the regulatory relationship between miR-135a-3p and ATM has been rarely reported. The current study demonstrated the targeting relationship between the two in T2D through a dual-luciferase gene reporter assay. ATM, which shares an association with T2D, regulates diabetogenic beta-cell survival in a P53-dependent manner (32). Besides, ATM protein was expressed in ɣ-H2AX-expressing testicular cells and could participate in histone phosphorylation in type 1 diabetes (33). Intriguingly, a higher nuclear DNA damage response was demonstrated in diabetic subjects with further accumulation of ATM (34). The knockout of ATM could attenuate endothelial dysfunction and cell senescence in the aorta of diabetic mice (35). It has been reported that an aberrant decline or loss of ATM was identified in ER/PR/ERBB2-triple-negative breast cancer (36).

Additionally, ATM regulated p53 stabilization in the somatic ErbB2 breast cancer model (37). Interestingly, an enhanced EGFR phosphorylation has been identified in diabetic vascular tissues collected from the constructed animal models and in human cells under conditions mimicking hyperglycemia (38, 39). Thus, we hypothesize that the activated ErbB signaling pathway exerts an inducing effect on vascular injury. Collectively, in our study, peripheral blood-derived Exos from T2D patients containing miR-135a-3p could aggravate vascular injury in T2D through the regulation of the ATM/ErbB axis.

## Conclusion

**Figure 9:**
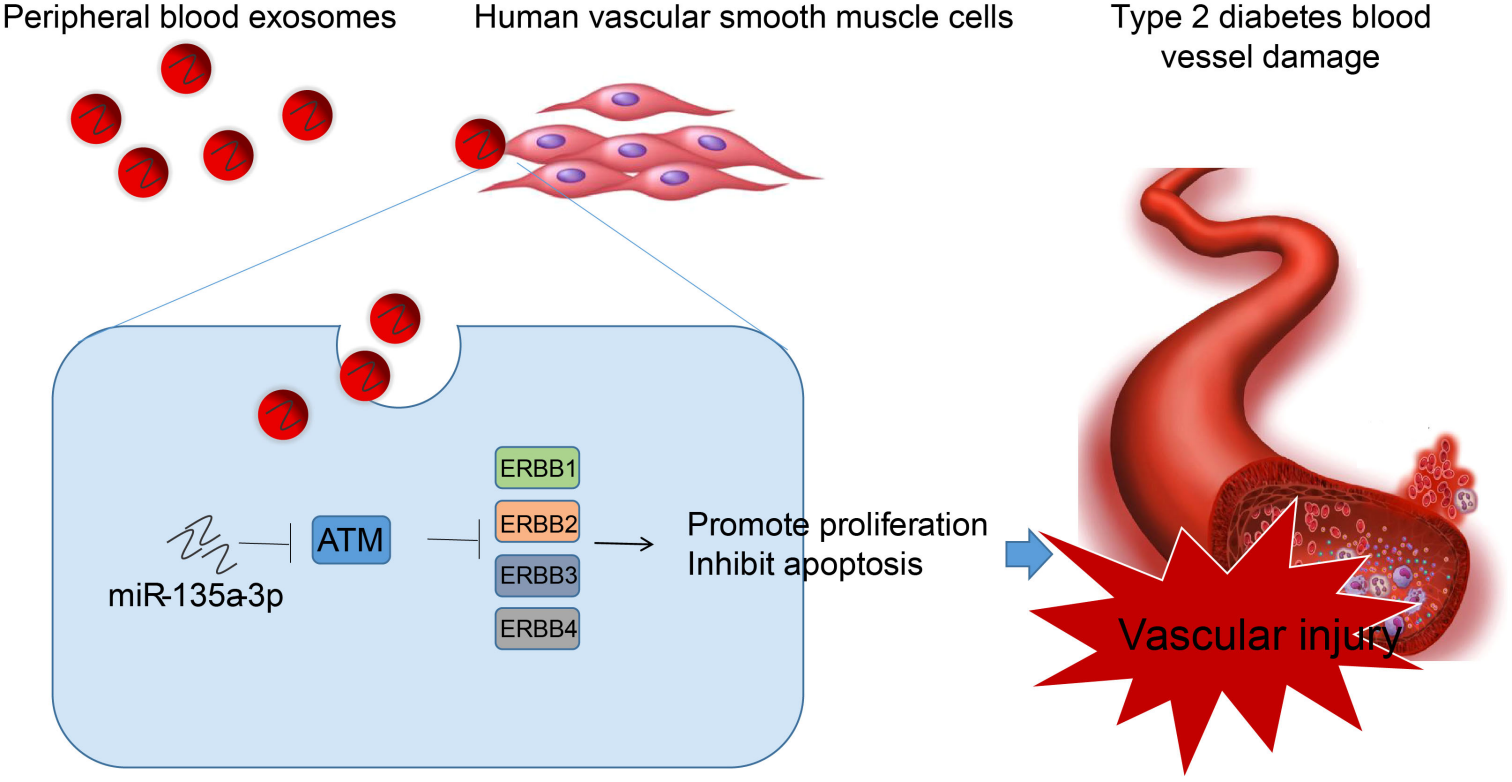
Molecular mechanism of peripheral blood-derived Exos containing miR-135a-3p in the occurrence of vascular injury in T2D through the ATM/ErbB axis. Peripheral blood-derived Exos from patients with T2D carry miR-135a-3p into HVSMCs, which inhibits ATM and activates the ErbB signaling pathway, thereby reducing vascular injury in T2D.

In summary, we concluded that peripheral blood-derived Exos from T2D patients carry miR-135a-3p into HVSMCs, which activates the ErbB signaling pathway by inhibiting ATM, thereby promoting vascular injury in T2D (**Figure 9**). This study screened T2D-related chip data from the GEO database and isolated peripheral blood exosomes, revealing significant upregulation of miR-135a-3p in T2D patient-derived exosomes. Through a series of analyses, including prediction of downstream targets of miR-135a-3p, construction of miR-135a-3p-regulated mRNA network relationship maps, GO and KEGG enrichment analysis, PPI network analysis, and luciferase experiments, we found that exosomes from peripheral blood carry miR-135a-3p into HVSMCs, which may target and inhibit ATM, activate the ErbB signaling pathway, and promote abnormal proliferation and migration of HVSMCs, leading to T2D vascular injury. This study reveals the molecular mechanisms underlying T2D vascular injury and provides new ideas and targets for further research on treating and preventing T2D vascular injury.

## Data availability statement

The original contributions presented in the study are included in the article/**Supplementary Materials**, further inquiries can be directed to the corresponding author/s.

## Ethics statement

The studies involving humans were approved by the Medical Ethics Committee, Xiangya Hospital, Central South University (202305356). The studies were conducted in accordance with the local legislation and institutional requirements. The participants provided their written informed consent to participate in this study.

## Author contributions

The research was designed by KX and YC. The peripheral blood samples were collected by CL and MW. The cell experiment was completed by KX and MW. The research data was colected and analysed by CL and SF. The manuscript was drafted by KX and YC. All authors contributed to the article and approved the submitted version.
