# Supplementary material for: miR-135a-5p overexpression in peripheral blood-derived exosomes mediates vascular injury in type 2 diabetes patients
Source: Front Endocrinol (Lausanne). 2023 Nov 3;14:1035029. doi: 10.3389/fendo.2023.1035029 (PMC10657216; doi:10.3389/fendo.2023.1035029)
Supplement: Supplementary file 1 [file Table_1.docx]

**Table S1** Primer sequences for RT-qPCR

| Gene | Primer sequence (5’-3’) |
| --- | --- |
| hsa-miR-1321 | CCCTGAGTTTATTTCCTTAT |
| hsa-miR-146b-3p | AACTGAGTCCACAGGGCATT |
| hsa-miR-422a | GAGACTCAGGCCTTCTGACC |
| hsa-miR-212-5p | GTGACTGGAGACTGTTACTGAGGG |
| hsa-miR-135a-3p | CCACGGCTCCAATCCCTATA |
| hsa-miR-92a-1 | AACTCAACAGGCCGGGACAA |
| hsa-miR-19b-2 | CATGGATTTGCACAGCCACA |
| hsa-miR-32-3p | ACACTAAATTGCATTGAGGC |
| hsa-miR-154-5p | TCAACCGTGTATGATTCGTC |
| hsa-miR-192-5p | AGGCGAACATACCTGTGACCTA |
| hsa-miR-363-3p | TTTACAGATGGATACCGTGC |
| hsa-miR-215 | ATATTGGCCTAAAGAAATGA |
| U6 | Forward: CTCGCTTCG GCAGCACA |
|  | Reserve: Universal reverse primer |

Note: miR, microRNA; RT-qPCR, reverse transcription-quantitative polymer chain reaction.
